# FOURIER & PCSK9 RNAi: Towards enhancing durability and efficacy of PCSK9 inhibitors

**DOI:** 10.21542/gcsp.2017.13

**Published:** 2017-06-30

**Authors:** Mohamed Hassan

**Affiliations:** Aswan Heart Centre, Aswan, Egypt

## Introduction

The 2003 discovery of proprotein convertase subtilisin–kexin type-9 (PCSK9) – a circulating protein targeting the low density lipoprotein (LDL) receptor for its degradation^1,2^ – paved the way for the development of several therapeutic approaches which inhibit the protein itself (by monoclonal antibodies) or its RNA (by RNA interference “RNAi”).^3^ These drugs are a very promising and attractive therapy for high-risk cardiovascular (CV) patients, patients with statin intolerance, and those with familial hypercholesterolemia.

PCSK9-targeted monoclonal antibodies (evolocumab, and alirocumab) have been shown to dramatically lower LDL-C levels by up to 60% when combined with a statin. Earlier studies demonstrated robust efficacy and favorable safety profile for this new class of drugs,^4–8^ and exploratory data from the Open-Label Study of Long-Term Evaluation against LDL Cholesterol (OSLER-2) and Long-term Safety and Tolerability of Alirocumab in High Cardiovascular Risk Patients with Hypercholesterolemia Not Adequately Controlled with Their Lipid Modifying Therapy (ODYSSEY) trials showed significant reductions in CV outcomes.^9,10^ Subsequently the Food and Drug Administration (FDA) has approved the use of evolocumab and alirocumab for the treatment of certain patients who are unable to get their LDL cholesterol (LDL-C) under control despite maximally tolerated statin therapy. However, the long-term effect of these drugs on clinical CV outcome is uncertain.

Inclisiran (ALN-PCSsc) is a novel, longer acting, synthetic small interfering RNA (siRNA) molecule that binds intracellularly to the RNA-induced silencing complex (RISC), enabling it to cleave messenger RNA (mRNA) molecules encoding PCSK9 specifically.^11,12^ This siRNA molecule is conjugated to triantennary N-acetylgalactosamine carbohydrates which facilitates its targeted delivery to hepatocytes through its binding to abundant liver-expressed asialo-glycoprotein receptors.^13^

Data from the eagerly anticipated PCSK9- targeted antibodies outcome study (Further Cardiovascular Outcomes Research with PCSK9 inhibition in Subjects with Elevated Risk “FOURIER” study), and the phase I study of RNAi therapeutic agent targeting PCSK9 have been recently released.

## FOURIER study

FOURIER study was a prospective, double-blind, placebo-controlled phase III study that has been recently presented in the American College of Cardiology (ACC) Scientific Sessions 2017, and simultaneously published in *the New England Journal of Medicine* journal.^14^ This study was designed to test the effect of evolocumab on primary endpoint of death, myocardial infarction (MI) and hospitalization . A total of 27,564 patients with atherosclerotic CV disease and LDL-C levels ≥ 70 mg/dl -despite maximally tolerated statin therapy- were randomly assigned to receive evolocumab (either 140 mg every 2 weeks or 420 mg monthly) or matching placebo as subcutaneous injections. The primary efficacy end point was the composite of CV death, MI, stroke, hospitalization, or coronary revascularization. The key secondary efficacy end point was the composite of CV death, MI, or stroke. The median duration of follow-up was 2.2 years.

**Figure 1.**
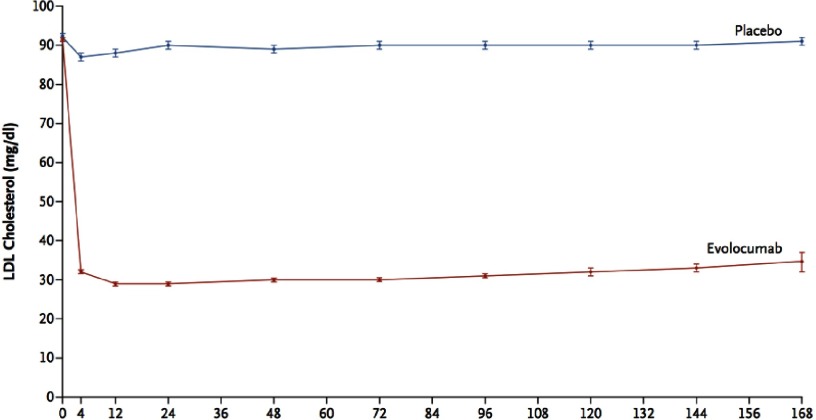
Effect of evolocumab, compared to Placebo, on LDL-C level over time.

**Figure 2.**
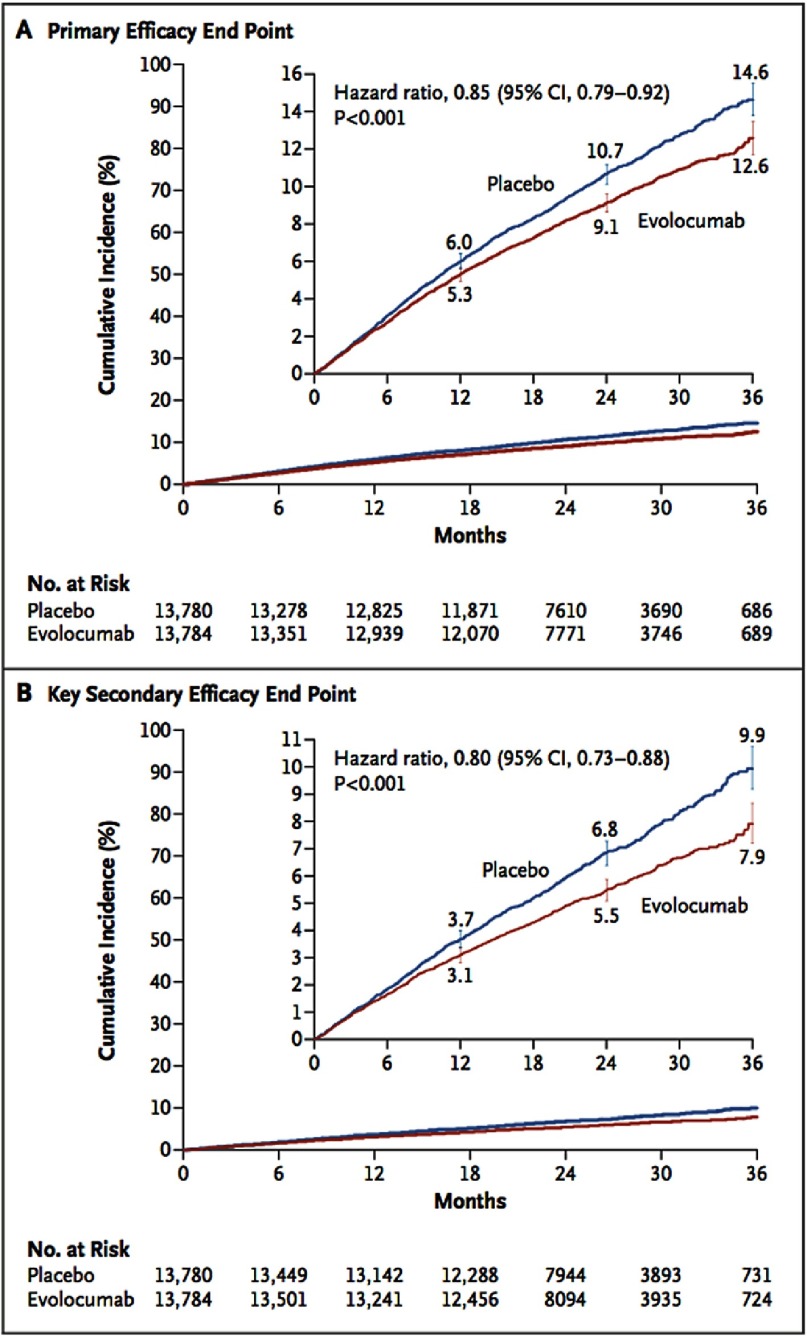
Incidence of cardiovascular events in patients received evolocumab compared to placebo.

At 48 weeks, the least-squares mean percentage reduction in LDL-C levels with evolocumab, as compared with placebo, was 59%, from a median baseline value of 92 mg/dL to 30 mg/dL (*P* < 0.001) (Figure 1). The primary end point was significantly reduced with evolocumab treatment compared to placebo (9.8% vs. 11.3%; hazard ratio 0.85; 95% confidence interval [CI], 0.79 to 0.92; P<0.001). The key secondary end point was also significantly reduced with evolocumab treatment compared to placebo (5.9% vs. 7.4%; hazard ratio, 0.80; 95% CI, 0.73 to 0.88; *P* < 0.001) (Figure 2). The results were consistent across all subgroups, including patients in the lowest quartile for baseline LDL-C levels (median LDL-C 74 mg/dL). There was no significant difference between the study groups with regard to adverse events (including new-onset diabetes and neurocognitive events). Injection-site reactions were more common with evolocumab (2.1% vs. 1.6%).

## The study of RNAi therapeutic agent targeting PCSK9

This was a phase 1 randomized clinical trial that has been recently published in the *New England Journal of Medicine*.^12^ The study was designed to evaluate the safety, side-effect profile, and pharmacodynamic measures (PCSK9 level, LDL-C level) of inclisiran. Healthy volunteers were randomly assigned with an LDL-C level ≤ 100 mg/dL in a 3:1 ratio to receive a subcutaneous injection of inclisiran or placebo in either a single-ascending-dose phase (at a dose of 25, 100, 300, 500, or 800 mg) or a multiple-dose phase (125 mg weekly for four doses, 250 mg every other week for two doses, or 300 or 500 mg monthly for two doses) with or without concurrent statin therapy. Each dose cohort included 4-8 participants.

**Figure 3.**
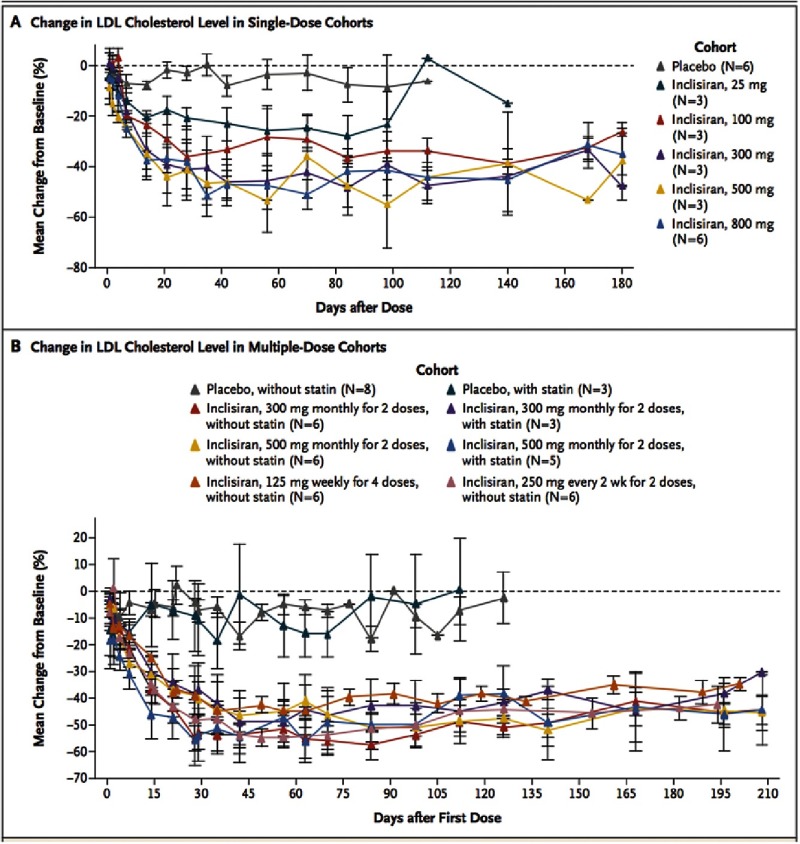
Effects of Inclisiran on Serum Levels of LDL- C, According to Study Group and Dose Cohort.

The most common adverse events were cough, musculoskeletal pain, nasopharyngitis, headache, back pain, and diarrhea. All adverse events were mild or moderate in severity. No serious adverse events or discontinuations due to adverse events were reported. Single inclisiran doses of 300 mg or more reduced the PCSK9 level (up to a least- squares mean reduction of 74.5% from baseline), and doses of 100 mg or more reduced the LDL cholesterol level (up to a least-squares mean reduction of 50.6% from baseline) (Figure 3). Reductions in the levels of PCSK9 and LDL-C were maintained at day 180 for doses of 300 mg or more. All multiple-dose regimens reduced the levels of PCSK9 (up to a least-squares mean reduction of 83.8% from baseline) and LDL-C (up to a least-squares mean reduction of 59.7% from baseline).

## Discussion

The results of these two studies add to the clinical evidence which supports PCSK9 as a therapeutic target for significant lowering of the LDL-C level in high CV risk patients. The addition of evolocumab to statin therapy was associated with 15% reduction in the risk of primary composite end point of CV death, MI, stroke, hospitalization for unstable angina, or coronary revascularization and a 20% reduction in the risk of the more clinically serious key secondary end point of CV death, MI, or stroke.^14^ Importantly, evolocumab did not affect memory or other cognitive problems—at least for up to 2 years, in the Evaluating PCSK9 Binding Antibody Influence On Cognitive Health in High Cardiovascular Risk Subjects (EBBINGHAUS) study, which has been recently presented in the ACC scientific sessions 2017. On the other hand, doses of 300 mg or more (in single or multiple doses) of inclisiran -the RNAi therapeutic agent targeting PCSK9- significantly reduced levels of PCSK9 and LDL-C for a longer time (up to 6 months) with no reported serious adverse events.

Lowering LDL-C level when ezetimibe is added to statin therapy in the Improved Reduction of Outcomes: Vytorin Efficacy International Trial (IMPROVE-IT) also had similar effects on reducing major CV events.^15^ This strongly reaffirms the LDL hypothesis that low is good, but lower is better.

Inclisiran differs in its mode of action and pharamcodynamic profile from anti-PCSK9 antibodies. Whereas anti- PCSK9 antibodies bind to extracellular PCSK9 (produced from any tissue) and prevent its interaction with the LDL receptor, inclisiran interferes with PCSK9 mRNA transcription and inhibits its synthesis specifically in the liver. In addition, the effect of inclisiran on PCSK9 and LDL-C levels persists for at least 6 months after the initiation of treatment, with little variation during this period.^11,12^

PCSK9 RNA interference by Inclisiran may provide long term and effective management of hypercholesterolemia with administration every 3 or 6 months, as compared with the once or twice monthly regimen for the currently approved anti-PCSK9 antibodies. However, the results need to be further validated in a large randomized clinical trial.

## What have we learned?

Significant reduction in the residual cardiovascular risk, despite optimal statins therapy, can be achieved by the new class of drugs that target PCSK9. After the results of the FOURIER study, international clinical guidelines may need to re-address their future recommendations - other than their current class IIb recommendations^16^ - regarding the use of PCSK9 inhibitors in high-risk patients with hypercholesteremia - also taking into consideration the fact that the cost of these drugs is currently prohibitively high.
